# NAA/Glu Ratio Associated with Suicidal Ideation in Pilot Sample of Autistic Youth and Young Adults

**DOI:** 10.3390/brainsci12060785

**Published:** 2022-06-15

**Authors:** Iska Moxon-Emre, Paul E. Croarkin, Zafiris J. Daskalakis, Daniel M. Blumberger, Rachael E. Lyon, Hideaki Tani, Peter Truong, Meng-Chuan Lai, Pushpal Desarkar, Napapon Sailasuta, Peter Szatmari, Stephanie H. Ameis

**Affiliations:** 1 Cundill Centre for Child and Youth Depression, The Margaret and Wallace McCain Centre for Child, Youth & Family Mental Health, Campbell Family Mental Health Research Institute, Centre for Addiction and Mental Health, Toronto, ON M5T 1R8, Canada; iska.moxon-emre@camh.ca (I.M.-E.); rachael.lyon@camh.ca (R.E.L.); htani.neuropsy@gmail.com (H.T.); mengchuan.lai@utoronto.ca (M.-C.L.); peter.szatmari@camh.ca (P.S.); 2 Department of Psychiatry and Psychology, Division of Child and Adolescent Psychiatry, Mayo Clinic, Rochester, MN 55905, USA; croarkin.paul@mayo.edu; 3 Temerty Centre for Therapeutic Brain Intervention, Campbell Family Mental Health Research Institute, Centre for Addiction and Mental Health, Toronto, ON M6J 1H1, Canada; zdaskalakis@health.ucsd.edu (Z.J.D.); daniel.blumberger@camh.ca (D.M.B.); pushpal.desarkar@camh.ca (P.D.); 4 Department of Psychiatry, Temerty Faculty of Medicine, University of Toronto, Toronto, ON M5T 1R8, Canada; 5 Research Imaging Centre, Centre for Addiction and Mental Health, Toronto, ON M5T 1R8, Canada; truonp@outlook.com (P.T.); pom4pom@gmail.com (N.S.); 6 Research Institute, Department of Psychiatry, The Hospital for Sick Children, Toronto, ON M5G 1X8, Canada; 7 Department of Psychology, University of Toronto, Toronto, ON M5S 3G3, Canada

**Keywords:** autism spectrum disorder, suicidality, magnetic resonance spectroscopy

## Abstract

Suicidality is increased in autism spectrum disorder (ASD), yet effective interventions are lacking. Developing biologically based approaches for preventing and treating suicidality in ASD hinges on the identification of biomarkers of suicidal ideation (SI). Here, we assessed magnetic resonance spectroscopy (MRS) markers of glutamatergic neurotransmission in ASD youth and young adults. Twenty-eight ASD participants (16–33 years) underwent ^1^H-MRS, and metabolites were quantified using LCModel. N-acetylaspartate (NAA), glutamate (Glu), and the NAA/Glu ratio from the left dorsolateral prefrontal cortex were compared between ASD SI+ (*n* = 13) and ASD SI− (*n* = 15) participants. We found that ASD SI+ participants had a higher NAA/Glu ratio compared ASD SI− participants. The NAA/Glu ratio also predicted SI and significantly discriminated between ASD SI+/SI− participants. All analyses including NAA and Glu alone were non-significant. Here, we provide preliminary evidence for the importance of NAA/Glu in ASD with SI, with implications for biomarker discovery. Further mechanistic research into risk and interventional approaches to address SI in ASD are needed.

## 1. Introduction

Suicidality in individuals diagnosed with autism spectrum disorder (ASD) is a global health concern. Recent meta-analytic evidence derived from North American, European, and Asian samples revealed children and adults with ASD are at increased risk of suicide [1]. While suicide is a leading cause of youth death in the general population [2,3], the even higher rates in ASD youth are a growing concern. Despite the urgent need to address this public health problem, studies that describe the phenomenology and biological associations of suicidality in ASD youth and young adults are currently lacking. Identifying neurobiological markers of suicidal ideation (SI) in ASD youth and young adults is also a critical first step for the development of biologically informed targeted interventions.

Disruptions in glutamate (Glu) excitatory neurotransmission and metabolism have been implicated in suicidality [4]. Evidence in this regard, however, is mixed [5]. Localized primarily to neurons, N-acetylaspartate (NAA) concentration is thought to reflect neuronal density [6]. Preliminary findings suggest NAA concentrations in the right dorsolateral prefrontal cortex (DLPFC) of depressed adults with suicidal behavior may be lower than that in a group of controls [7]. Notably, NAA can be converted to Glu [8]. Given this shared metabolic pathway, it may be valuable to consider NAA and Glu together as a dynamic metabolic measure when examining complex symptom dimensions such as suicidality.

Preliminary evidence suggests that glutamatergic markers may also be promising targets for identifying adolescents with SI. Lewis and colleagues [9] found that proton magnetic resonance spectroscopy (^1^H-MRS) measures of the NAA to Glx (Glx = glutamate + glutamine) ratio (NAA/Glx) in the anterior cingulate cortex (ACC) discriminated between depressed adolescents with and without SI. Imbalanced excitatory–inhibitory neurotransmission is a hypothesized etiological factor in ASD [10,11,12]. Thus, ^1^H-MRS derived glutamatergic markers could prove useful in understanding mechanisms and perhaps as a marker of treatment response for future studies of SI and its treatment in ASD youth and young adults.

Here, we undertook a preliminary evaluation of ^1^H-MRS-measured glutamatergic markers from the left DLPFC (MRS data were not collected from the right DLPFC), in a small convenience sample of ASD youth and young adults. We hypothesized that ASD participants with SI, compared to those without SI, would have higher NAA/Glu levels. We further hypothesized that NAA/Glu would predict SI, and discriminate between participants with and without SI.

## 2. Results

Thirteen participants in our sample endorsed suicidal thinking (SI+ group), and fifteen participants did not (SI− group), as characterized in Table 1. The SI+/SI− groups did not differ on demographic characteristics. The SI+ group contained more depressed participants (classified using the Mini International Neuropsychiatric Interview [MINI]) than the SI− group (*p* = 0.03, Fisher’s exact). The SI+ group also had more participants with additional co-occurring mental health conditions compared to the SI− group (*p* = 0.002, Fisher’s exact). Of note, a single participant with depression was in the SI− group.

**Figure 1:**
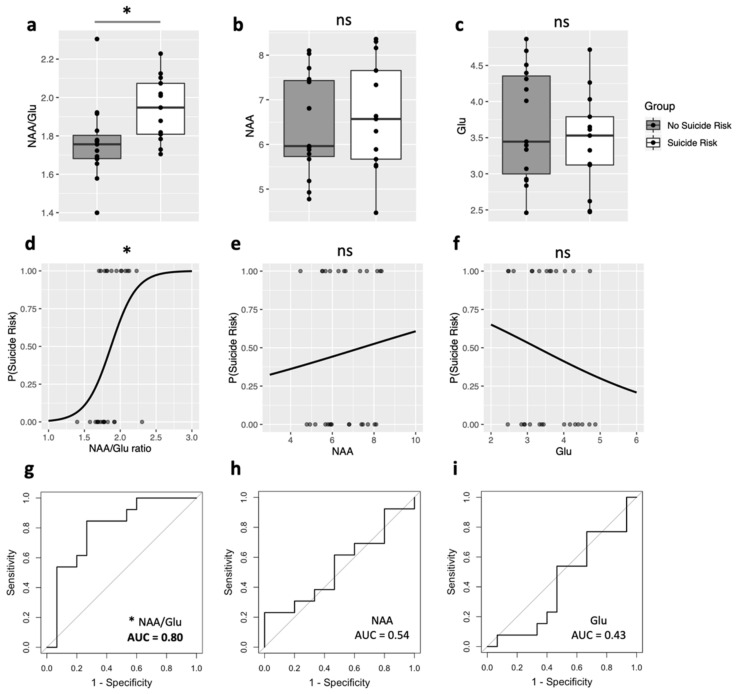
(**a**–**c**) Left dorsolateral prefrontal cortex (L-DLPFC) metabolites measured with proton magnetic resonance spectroscopy (^1^H-MRS) in ASD participants with suicidal ideation (SI+; *n* = 13) and without (SI−; *n* = 15): (**a**) NAA/Glu: SI+ mean (SD) = 1.94 (0.17), SI− mean (SD) = 1.76 (0.20), * *p* < 0.05; (**b**) NAA: SI+ mean (SD) = 6.65 (1.24), SI− mean (SD) = 6.43 (1.12); (**c**) Glu: SI+ mean (SD) = 3.44 (0.68), SI− mean (SD) = 3.69 (0.77); (**d**–**f**) logistic regression models for the prediction of SI+ as a function of L-DLPFC metabolites, * *p* < 0.05; (**g**–**i**) receiver operating characteristic (ROC) curves of the discriminatory capacity for L-DLPFC metabolites to predict SI+. Area under the curve (AUC) prediction accuracy; a value of 1 indicates perfect accuracy whereas a value of 0.5 (grey diagonal line) indicates chance accuracy.

Participants in the SI+ group had higher NAA/Glu ratio than participants in the SI− group (F (_2,25_) = 6.19, *p* = 0.02, Cohen’s f = 0.50; Figure 1a). In contrast, the SI+ and SI− groups did not differ in NAA (F (_2,25_) = 0.28, *p* = 0.60, Cohen’s f = 0.11; Figure 1b) or Glu (F (_2,25_) = 0.89, *p* = 0.36, Cohen’s f = 0.19; Figure 1c).

Logistic regression revealed the NAA/Glu ratio significantly predicted SI, b = 0.56, SE = 0.27, z (25) = 2.11, *p* = 0.04, odds ratio = 1.76:1 (Figure 1d), implying that every 0.1-unit increase in NAA/Glu ratio predicted a 1.76 fold increase in the odds of SI. NAA did not predict SI, b = 0.02, SE = 0.04, z (25) = 0.49, *p* = 0.63, odds ratio = 1.02:1 (Figure 1e), nor did Glu b = −0.06, SE = 0.06, z (25) = −1.02, *p* = 0.31, odds ratio = 0.94:1 (Figure 1f).

Sensitivity and specificity estimates for separating ASD SI+ from SI− participants were obtained with ROC analyses. AUC analyses indicated that NAA/Glu in the left DLPFC discriminated between ASD SI+ and ASD SI− participants (AUC = 0.8; SE = 0.09; 95% confidence interval [CI] = 0.63–0.97; Figure 1g); neither NAA (AUC = 0.54; SE = 0.12; 95% CI = 0.32–0.77; Figure 1h), nor Glu alone (AUC = 0.43; SE = 0.11; 95% CI = 0.20–0.65; Figure 1i) discriminated ASD SI+ from ASD SI− participants. A cutoff point of 1.78 for NAA/Glu yielded a sensitivity of 0.85 and a specificity of 0.80 for discriminating between the presence/absence of SI in ASD.

## 3. Discussion

Here, we assessed ^1^H-MRS-measured markers of glutamatergic neurotransmission from the left DLPFC in a sample of ASD youth and young adults. Our findings provide preliminary evidence for the potential importance of NAA/Glu in ASD with SI. We found that ASD SI+ participants had a higher NAA/Glu ratio compared to ASD SI− participants. NAA/Glu also predicted SI and significantly discriminated between ASD SI+ and ASD SI− participants.

Our findings are partially consistent with Lewis et al. (2020), where NAA/Glx was found to discriminate SI+ from SI− adolescents with depression. Though broadly similar, the direction of our results differ from Lewis et al. (2020) in that NAA/Glu was higher in ASD SI+ participants in our sample, whereas NAA/Glx was lower in SI+ adolescents with depression in their sample. Several key sample and methodological differences may contribute to the discrepant finding. We included ASD youth and young adults with and without depression, whereas participants reported on in Lewis et al. (2020) were adolescents with depression without ASD. Given the emerging evidence for altered excitatory–inhibitory neurotransmission in ASD [10,11,12]), combined with neurometabolite alterations documented in depression [13], it is perhaps unsurprising that our findings do not perfectly align. We also examined MRS-measured metabolites from a left DLPFC voxel, whereas an ACC voxel was used in Lewis et al. (2020). Moreover, owing to acquisition sequence differences (MEGA-PRESS vs. J-PRESS), we assessed the NAA/Glu ratio, whereas Lewis et al. (2020) assessed the NAA/Glx ratio. Glu and Glx are not identical markers, given that Glx encompasses both Glu and glutamine signals [6]. Nonetheless, our collective findings suggest that NAA/Glu and NAA/Glx warrant further investigation as candidate biomarkers associated with SI.

Prior work has suggested that the ratio of NAA to glutamatergic metabolites may be more sensitive for capturing metabolic alterations than either metabolite alone [9,14]. It is notable that we found ASD SI+ participants to have a higher NAA/Glu ratio in the absence of detectable differences in NAA or Glu between ASD SI+/SI− groups. Though speculative, this finding may reflect dysregulated NAA to Glu metabolism in ASD SI+ youth and young adults. Given that NAA can be converted to Glu, especially under conditions of metabolic stress [8], disrupted conversion of NAA to Glu could conceivably result in higher NAA and lower Glu concentrations that are indetectable across ASD SI+/SI− groups, but that manifest as detectable differences in their ratio across groups.

Reliable biomarkers of SI in ASD youth and young adults may help identify at-risk individuals, with plausible utility for treatment planning (i.e., predicting potential for treatment response) and tracking neurobiological responses to interventions. We recently found that bilateral rTMS to DLPFC modulates Glx levels in the left DLPFC (MRS data were not collected from the right DLPFC) and that baseline Glx predicted change in Glx from pre- to post-rTMS in the same ASD sample included here [15]. There is evidence that excitatory rTMS alters the glutamatergic system [16,17,18] and may normalize NAA levels in treatment-resistant depression [19]. Consequently, rTMS intervention studies that track changes to glutamatergic markers and suicidality symptoms concurrently may help establish whether rTMS yields clinical improvement by altering glutamatergic neurotransmission.

The present study has a number of limitations. The convenience sample reported on here was small (*n* = 28), thus we did not evaluate the potential role of key demographic and clinical characteristics (e.g., sex, medication use) on our NAA/Glu findings. Studies with larger sample sizes that are designed and powered to examine the relationship between SI and NAA/Glu are required to replicate the preliminary results reported here. Further, as our sample included depressed and non-depressed participants, future studies conducted with an exclusively depressed sample of ASD youth and young adults are needed to clarify the unique and/or shared role of neurometabolites in depression +/− suicidality. Given we excluded participants with active suicidal intent, our findings are not representative of the entire spectrum of suicidality. Additionally, MRS data were collected from the left DLPFC despite bilateral stimulation. MRS data should be acquired bilaterally in future rTMS studies. Finally, there are a number of limitations inherent to ^1^H-MRS, which we have detailed extensively in Moxon–Emre et al. (2021).

## 4. Materials and Methods

*Participants:* Twenty-eight ASD youth and young adults (mean (SD) = 23.3 (4.69) years, range 16–33 years, 21 male/7 female assigned at birth) underwent ^1^H-MRS scans during a baseline assessment of our recently completed repetitive transcranial magnetic stimulation (rTMS) clinical trial (Clinicaltrials.gov; ID: NCT02311751) [15,20,21]. The present sample is identical to the baseline ASD sample from Moxon–Emre et al. (2021), wherein we detail specifics pertaining to final sample selection, including quality control (QC) of ^1^H-MRS data used here. Full clinical trial details, including complete inclusion/exclusion criteria, have also been reported previously [20]. Briefly, participants aged 16–35 years with a prior DSM-5 diagnosis of ASD and confirmed using the Autism Diagnostic Observation Schedule-2 (ADOS-2) [22], with clinically significant executive dysfunction (based on any Behavior Rating Inventory of Executive Function-Adult [BRIEF-A] [23] subscale T score > 65) and without intellectual impairment (IQ ≥ 70 on the General Abilities Index (GAI) from the Wechsler Adult Intelligence Scale—Fourth Edition (WAIS-IV) [24], were recruited either from mental health clinics at the Centre for Addiction and Mental Health (CAMH, Toronto, Canada), community clinics, or through local advertisements. Participants were excluded if they had prior major medical or neurological illnesses, were taking anticonvulsants or benzodiazepines (≥2 mg lorazepam equivalent), or had a history of substance abuse/positive toxicology screen. Participants with active suicidal ideation (e.g., with a suicidal plan and/or intent) that were deemed to be clinically unstable (based on clinical assessment by a study psychiatrist SHA, MCL, or PD) were also excluded. This study was approved by the research ethics board at CAMH, and all participants provided written informed consent prior to clinical trial enrollment.

*Measures:* Co-occurring mental health conditions were assessed using the Mini International Neuropsychiatric Interview (MINI) for participants aged ≥ 18 years [25], and the MINI for children and adolescents (MINI-KID) for participants aged 16–17 [26]. The MINI is a structured interview for diagnosing DSM-IV disorders and suicide risk was assessed using the suicidal tendency module. Participants who answered “yes” to any of the module’s nine questions were included in the SI+ group, and all others were included in the SI− group. All other measures administered as part of this clinical trial are detailed in Ameis et al., (2017).

*Magnetic Resonance Spectroscopy*: ^1^H-MRS data were acquired on a 3 Tesla GE MR750 (General Electric, Milwaukee, WI) scanner using a MEshcher–Garwood Point RESolved Spectroscopy (MEGA-PRESS) sequence (TE = 68 ms, TR = 1500 ms, 512 averages), from a 20 × 40 × 30 mm^3^ voxel in the left DLPFC. The left DLPFC was selected for MRS voxel placement as imaging time constraints in our clinical trial prevented bilateral MRS data acquisition, and rTMS studies that include MRS data have mainly acquired data from a left hemisphere voxel [16,18,27,28,29,30,31]. Complete MRS data acquisition, processing, and structural imaging details for our sample are provided in Moxon–Emre et al., (2021). Briefly, the GANNET 3.0 [32] processing pipeline was used, the editing-OFF raw data was separated out using the FID-A toolkit [33], and metabolites were quantified using LCModel version 6.3–0E [34]. The linewidth (full width at half maximum [FWHM]) of the water reference was measured, and <10 Hz was required for inclusion. For our MRS sequences, the mean (SD) signal-to-noise ratio (SNR) was 41.5 (6.58), and the mean (SD) linewidth was 8.71 (0.94). Quality of spectral fitting was assessed using the standard Cramer–Rao lower bound (CRLB) values, and fitted spectra with CRLB < 20% were included (%SD for Glu: mean = 7.18 ± 2.23 and %SD for NAA: mean = 2.29 ± 0.54). We corrected water-scaled metabolite concentrations for voxel tissue composition.

*Statistical analyses:* Demographic and clinical characteristics of the SI+ and SI− groups were compared using Mann–Whitney *U* tests for continuous variables, and Fisher’s exact tests for categorical variables. To test our first hypothesis, NAA/Glu, NAA, and Glu levels were compared between SI+/SI− groups, using ANCOVAs covarying for age. To test our second hypothesis, three separate logistic regressions were used to model SI as a function of NAA/Glu, NAA, and Glu levels, controlling for age. Receiver operating characteristic (ROC) analyses were conducted to assess the discriminatory capacity of NAA/Glu, NAA, and Glu levels for predicting SI. The area under the curve (AUC) was tested against an AUC of 0.5. ROC analyses were conducted using the pROC package in R, version 1.16.2 [35].

## Figures and Tables

**Table 1:** Sample demographic and clinical characteristics.

	Suicidal Ideation (SI+)	No Suicidal Ideation (SI−)	
	(*n* = 13)	(*n* = 15)	*U*
** Age **			
Mean (SD)	23.4 (5.19)	23.1 (4.39)	
Median [Min, Max]	25.0 [16.0, 33.0]	21.0 [17.0, 31.0]	0.98
** Sex **			
Number of males (%)	8 (61.5%)	13 (86.7%)	0.20
Psychotropic Medication *			
Number of participants (%)	7 (53.8%)	10 (66.7%)	0.70
** MINI **			
Comorbidity—other than suicide **			
** ** Number of participants (%)	11 (84.6%)	3 (20.0%)	**0.002**
Suicide risk level ***			
** ** Low	7 (53.8%)	-	
** ** Moderate	5 (38.5%)	-	
** ** High	1 (7.7%)	-	
** Depression, current (2 weeks) **			
** ** Number of participants (%)	6 (46.2%)	1 (6.7%)	**0.03**
** Depression, recurrent **			
** ** Number of participants (%)	2 (15.4%)	0 (0%)	0.21
** Years of Education **			
Mean (SD)	13.4 (2.40)	14.9 (3.37)	0.24
Median [Min, Max]	13.0 [10.0, 17.0]	14.0 [11.0, 22.0]	
** IQ—General Abilities Index **			
Mean (SD)	113 (17.9)	111 (18.3)	
Median [Min, Max]	113 [77.0, 140]	104 [79.0, 141]	0.70
** BRIEF Metacognition Index **			
Mean (SD)	70.2 (7.76)	71.0 (8.83)	
Median [Min, Max]	69.0 [59.0, 84.0]	68.0 [59.0, 84.0]	0.89
** BRIEF Global Composite **			
Mean (SD)	67.3 (8.64)	68.7 (8.00)	0.70
Median [Min, Max]	67.0 [52.0, 82.0]	66.0 [60.0, 86.0]	
** Adaptive Functioning Composite **			
Mean (SD)	75.9 (12.3)	75.2 (7.61)	0.87
Median [Min, Max]	79.0 [58.0, 104]	73.0 [61.0, 89.0]	

* Psychotropic medications were comparable across SI−/SI+ groups, which included: selective serotonin reuptake inhibitors (SSRI) (SI− *n* = 5/SI+ *n* = 5), selective norepinephrine reuptake inhibitors (SNRI) (SI+ *n* = 1), tetracyclic antidepressants (TCA) (SI− *n* = 1), norepinephrine dopamine reuptake inhibitors (NDRI) (SI− *n* = 1/SI+ *n* = 1), atypical antipsychotics (SI− *n* = 2/SI+ *n* = 2), amphetamines (SI− *n* = 1), methylphenidate (SI− *n* = 4), benzodiazepines (SI− *n* = 1/SI+ *n* = 1), and medical marijuana (SI− *n* = 1). ** Comorbidities include: major depressive episode, hypomanic episode, panic disorder, agoraphobia, generalized social phobia, obsessive compulsive disorder, psychotic disorders, mood disorder with psychotic features, generalized anxiety disorder, anorexia nervosa, and antisocial personality disorder. Suicide was excluded on the basis of it being used as our SI+/SI− grouping variable. *** Suicide risk level corresponds to risk categories from the MINI suicidality module (low risk = 1–8 points; moderate risk = 9–16 points; high risk ≥ 17 points). Age, years of education, IQ, BRIEF and Adaptive Function scores were compared between groups using Mann–Whitney U tests. All other (categorical) measures were compared using Fisher’s exact tests, given the small cell counts. BRIEF: Behavior Rating Inventory of Executive Function; MINI: Mini International Neuropsychiatric Interview; adaptive functioning composite from the Vineland Adaptive Behavior Scale-II (VABS-II).

## Data Availability

The datasets presented in this article are not readily available because we do not have consent to share this data.
